# Glucocorticoids and Glucocorticoid-Induced-Leucine-Zipper (GILZ) in Psoriasis

**DOI:** 10.3389/fimmu.2019.02220

**Published:** 2019-09-13

**Authors:** Lisa M. Sevilla, Paloma Pérez

**Affiliations:** Animal Models of Skin Pathologies Unit, Instituto de Biomedicina de Valencia (IBV)-CSIC, Valencia, Spain

**Keywords:** glucocorticoids (GCs), glucocorticoid-induced-leucine-zipper (GILZ/TSC22D3), skin inflammation, psoriasis, keratinocytes, immune cells, signaling

## Abstract

Psoriasis is a prevalent chronic inflammatory human disease initiated by impaired function of immune cells and epidermal keratinocytes, resulting in increased cytokine production and hyperproliferation, leading to skin lesions. Overproduction of Th1- and Th17-cytokines including interferon (IFN)-γ, tumor necrosis factor (TNF)-α, interleukin (IL)-23, IL-17, and IL-22, is a major driver of the disease. Glucocorticoids (GCs) represent the mainstay protocol for treating psoriasis as they modulate epidermal differentiation and are potent anti-inflammatory compounds. The development of safer GC-based therapies is a high priority due to potentially severe adverse effects associated with prolonged GC use. Specific efforts have focused on downstream anti-inflammatory effectors of GC-signaling such as GC-Induced-Leucine-Zipper (GILZ), which suppresses Th17 responses and antagonizes multiple pro-inflammatory signaling pathways involved in psoriasis, including AP-1, NF-κB, STAT3, and ROR-γt. Here we review evidence regarding defective GC signaling, GC receptor (GR) function, and GILZ in psoriasis. We discuss seemingly contradicting data on the loss- and gain-of-function of GILZ in the imiquimod-induced mouse model of psoriasis. We also present potential therapeutic strategies aimed to restore GC-related pathways.

## Introduction

Endogenous glucocorticoids (GCs) regulate development, metabolism, and immune responses in mammals (1, 2). In healthy individuals, GCs are synthesized by the adrenal glands and released to circulation as the final step of a complex cascade governed by the central hypothalamic–pituitary–adrenal (HPA) axis, with key roles in basal, and stress-related homeostasis (2). In addition, GCs can be produced locally by multiple tissues including the nervous system, thymus, and epidermis (3, 4). Synthetic GC counterparts are widely used as the first and most effective treatment to combat acute and chronic inflammatory pathologies. Both endogenous and exogenous GCs exert their actions through binding to the GC receptor (GR/*NR3C1*), a protein of the superfamily of nuclear hormone receptors that act as ligand-regulated transcription factors (5).

A main mechanism of GR action involves binding to genomic regulatory sequences called GR response elements to induce or repress target gene expression. GR induces genes encoding for anti-inflammatory mediators such as GC-Induced-Leucine-Zipper (GILZ), Dual-Specificity protein Phosphatase 1 (DUSP1), Inhibitor of kappaB alpha (IκBα), and Zinc Finger Protein 36/TrisTetraProlin (ZFP36/TTP) (6). Also, GR represses pro-inflammatory genes induced by the NF-κB and Mitogen Activated Protein Kinase (MAPK)/AP-1 pathways through protein-protein interactions that do not require GR binding to DNA. These two mechanisms classically referred to as transactivation and transrepression, respectively, coexist and are required for the optimal anti-inflammatory actions of GCs. Notwithstanding their effectivity, GC-based therapy is accompanied by side-effects of variable severity (the most extreme including metabolic syndrome, osteoporosis, and impairment of childhood growth), which may advise to discontinue treatment (7).

GILZ (encoded by the *TSC22D3* gene) was identified more than 20 years ago as anti-apoptotic in dexamethasone-treated thymocytes (8). Since then, GILZ expression has been reported in cell types of immune, and non-immune lineages. Multiple GILZ isoforms, resulting from alternative transcriptional initiation and splicing, have been identified with differential activities, and tissue specific expression patterns (9, 10). As of now, the majority of studies regarding therapeutic applications have been centered on the *GILZ1* isoform (referred to as *GILZ* hereafter). GILZ plays an anti-inflammatory role in macrophages, is crucial to regulate proliferation, survival, and differentiation in regulatory T (Treg) and dendritic cells; and contributes to regulation of phagocytosis in neutrophils and macrophages, thus putting an additional brake on chronic inflammation (11–14). GILZ is also expressed in airway epithelial cells (15), as well as in epidermal keratinocytes. In keratinocytes, GILZ is rapidly induced by GCs although its role in this cell type is not yet clarified (16–18).

GC immunosuppressive effects are exerted upon almost all immune cells including distinct effector lineages of T helper (Th) cells: Th1, Th2, Th17, or regulatory T (Tregs) (19). GCs inhibit Th1 development and induce differentiation of Th2 and Treg cells that limit immune response (20, 21). Th17 cells, producing interleukin 17 (IL-17) as their signature cytokine, are critical mediators of immune and inflammatory diseases including rheumatoid arthritis, asthma, and psoriasis (22). One key finding was the demonstration that GILZ increased Treg cell production by enhancing the transforming growth factor (TGF)-β/SMAD2 signaling pathway leading to induction of Foxp3, a lineage specific transcription factor responsible for development and function of these cells (21). GILZ has been shown to limit pro-inflammatory Th17 cell differentiation by binding to promoter regions and inhibiting expression of key cytokines, and classic Th17 transcription factors, like STAT3, and the master regulator of this cell lineage, retinoic acid-related orphan receptor (ROR)-γt (23).

Other anti-inflammatory GILZ actions are mediated through protein-protein interactions with NF-κB and AP-1 transcription factors precluding nuclear translocation, DNA binding, and regulation of gene expression (24, 25). Also, GILZ can bind to RAS/RAF, and thus suppress the MAPK pathway by inhibiting MAP2K/ERK1/2 phosphorylation (26).

*In vitro* studies in various cell types, including keratinocytes, showed GILZ downregulation upon treatment with pro-inflammatory mediators that activate toll-like receptors (TLRs) or cytokines such as tumor necrosis factor (TNF)-α, IL-1-β, or interferon (IFN)-γ (12, 15, 16, 27). In several chronic inflammatory diseases, GILZ expression inversely correlates with disease severity, suggesting that lower levels may aggravate these diseases and/or may be part of the pathogenesis [reviewed in (25, 28)] For instance, GILZ expression negatively correlates with disease severity in lupus patients, and murine models of this disease (29, 30). Moreover, *GILZ* mRNA was downregulated in white blood cells of sepsis patients (14), in activated macrophages of individuals with Crohn's disease (31), in patients with chronic rhinosinusitis where more pronounced decreases of *GILZ* associated with poor response to surgery (32), and in human psoriatic lesions (33, 34). However, in other instances, such as in the synovium of patients with active rheumatoid arthritis, GILZ levels were increased relative to healthy subjects; nevertheless, among patients being treated with therapeutic GCs, those able to induce GILZ showed improved disease activity (35). Overall these data underline that GILZ levels and activity are likely dependent on the disease type and tissue context.

## Mouse Models of Inflammation to Assess GILZ Function

GILZ was initially postulated as an alternative to GC therapies that could mediate GC immune-suppressive actions and anti-inflammatory effects without producing GC-associated side effects (11, 12, 25, 36). GILZ-deficient mice were viable and featured alterations that included male infertility due to impaired spermatogenesis, and electrolyte alterations (37–41). The lack of GILZ neither altered the immune response in several diseases (including arthritis and LPS-induced sepsis) nor decreased the anti-inflammatory effects of GCs in these models (37, 39, 42). Given that global GILZ-deficient mice had increased levels of endogenous GCs and other anti-inflammatory mediators, it is feasible that these compensatory mechanisms account for the observed results *in vivo* (28, 39, 43). In turn, the use of cell-type specific GILZ KO mouse models, such as macrophage-specific GILZ KO, which did not exhibit differences in their serum corticosteroid levels, represent a more adequate setting to investigate the impact of ablating endogenous GILZ (44).

However, in other settings, downregulation of GILZ during inflammation led to enhanced pro-inflammatory responses (44). For instance, the administration of GILZ siRNA enhanced disease progression in a mouse model of rheumatoid arthritis (45) and conversely, injection of GILZ-adeno-associated virus into the joints inhibited disease development to a similar extent as GC treatment (39). GILZ knockdown also resulted in increased disease severity in a mouse model of colitis due to pronounced granulocytic infiltrates and enhanced inflammation (13). GILZ-deficient macrophages showed increased responsiveness toward LPS, with augmented expression of pro-inflammatory cytokines due to ERK activation, and reduced desensitization to LPS, i.e., endotoxin tolerance (28).

In most mouse models of disease, higher levels of GILZ were protective against inflammation although with a variable degree of efficacy. The increased expression of GILZ in the SPRET/Ei mouse strain was shown to be the cause of its resistance to LPS-induced endotoxemia (46). GILZ overexpression with a T cell lineage specific promoter induced an anti-inflammatory Th2-type response in naive CD4 T cells (47), and these mice were less susceptible to a spinal cord injury model (48). Moreover, the use of GILZ peptides suppressed inflammation in a mouse model of autoimmune encephalomyelitis (24). Similarly, mice with generalized overexpression of GILZ (GILZ-Tg) had better survival rates in the cecal ligation and puncture sepsis model relative to controls (14). However, in this model, the protective effects of GILZ were not due to a decrease in systemic inflammation but linked to increased bacterial clearance due to more efficient phagocytosis by CD45^+^ peritoneal cells. Overall GILZ gain- or loss-of-function in mouse models of inflammation does not always result in opposite phenotypes. The cell-type specific mechanisms by which GILZ modulates tissue function both in normal homeostasis as well as in inflammatory settings need to be considered. The pleiotropic effects of GCs are mediated by numerous downstream targets in addition to GILZ; this biological redundancy likely accounts for the findings that GILZ deficiency does not always cause major inflammatory phenotypes.

## Psoriasis

The epidermis is composed of keratinocytes which terminally differentiate to form a permeability barrier essential for survival. The balance between keratinocyte proliferation and differentiation is tightly regulated, with alterations that affect barrier function leading to common inflammatory skin pathologies (49). One such disease, psoriasis, is a chronic relapsing inflammatory condition identified in 1–2% of the population, whose clinical presentation includes different symptoms and severity, age of onset, and location of skin lesions (50). Psoriatic patients typically develop reddish scaly plaques, and one-third of patients also have affected joints, which may lead to severe joint destruction (psoriatic arthritis). In addition, this disease shows high comorbidity with other inflammatory conditions such as metabolic and cardiovascular diseases (51).

Psoriasis pathophysiology is complex and includes both genetic and environmental risk factors. Dysregulation of Th1 and Th17 lineages leads to overproduction of various cytokines including IFN-γ, TNF-α, IL-23, IL-17, and IL-22 resulting in epidermal hyperproliferation and skin immune infiltrates (52). ROR-γt is induced during early Th17 differentiation and is a central driver of the later stages of this process (53). ROR-γt is present in IL-17-producing Th17 cells in a mouse model of psoriasis, indicating involvement in the disease, and is currently being investigated as a therapeutic target for drug design (54, 55). Both keratinocyte and lymphocytes can mediate psoriasis due to alterations in pro-inflammatory signaling pathways and transcription factors AP-1 [loss of function; (56–58)], as well as NF-κB, STAT3, and TGF-β [gain of function; (59, 60)].

Histopathological characterization of psoriatic lesions reveals epidermal thickening, abnormal epidermal differentiation, and increased epidermal protrusions (rete-ridges), along with intra-epithelial neutrophil infiltrates (Munro-like abscesses), and pronounced immune infiltrates consisting of T cells and dendritic cells (52). A widely used mouse model of psoriasis consists of topical applications of imiquimod, a TLR7 agonist, which induces the IL-23–Th17-cell axis and closely recapitulates the histopathological, and molecular characteristics of the human disease (57, 61, 62).

### Therapeutic Actions of Glucocorticoids

The symptoms of psoriatic patients can be treated systemically, topically, or by ultraviolet (UV) phototherapy (63). Classic treatments include synthetic compounds (GCs, retinoids, vitamin D derivatives, methotrexate, and cyclosporine) while novel therapies use antibodies targeting major cytokines associated with the disease (TNF-α, IL-17, and IL-23). As psoriasis is a relapsing disease, most patients require long-term management, which represents an important limitation for many of these treatments due to poor tolerability and/or cumulative toxicity (methotrexate and cyclosporine), or increased risk of non-melanoma skin cancer (phototherapy or TNF inhibitors) (64). These issues—age, specific symptoms, extent of lesions, and previous records of diseases—need to be addressed in the clinical practice to design efficient and safe treatments. While TNF-α and IL-17 inhibitors avoid many adverse effects of classic drugs, there are also concerns as these therapies can increase the risk of systemic infections, and their long-term use may represent an economic burden (52, 63).

GCs still represent the mainstay protocol for treating psoriatic patients with mild disease severity, and are preferably administered topically to minimize adverse side effects, including skin atrophy, loss of skin barrier function, increased susceptibility to infections, and delayed wound healing (65). However, in the long term, even topical GCs can cause Cushing's syndrome, and adrenal insufficiency with serious consequences (66). In addition, psoriatic patients with initially good responses to GCs can experience flares due to insensitivity to topical steroids (67). Downstream anti-inflammatory GC effectors such as ZFP36 and GILZ are attractive therapeutic candidates (36, 42, 68). Indeed GILZ is ideal as it interferes with multiple levels of pro-inflammatory signaling, including pathways involved in psoriasis like AP-1, NF-κB, STAT3, and ROR-γt. However, given the tissue- and cell type-specific differences in GILZ action it is important to decipher the impact of therapeutic doses of GILZ not only on skin immune cells, but also on epidermal keratinocytes, and dermal fibroblasts.

### Impaired Glucocorticoid-Signaling in Psoriasis

GCs limit skin inflammation by signaling through GR (69). Consistent with this, GR^−/−^ mice featured dramatically impaired epidermal differentiation, with decreased expression of differentiation markers, common features in human psoriasis (49, 70). Also, late embryos and newborn mice with epidermal-specific inactivation of GR featured phenotypic and molecular alterations similar to those observed in psoriasis, including enhanced expression of pro-inflammatory markers (71). However, these alterations resolved spontaneously by yet uncharacterized mechanisms, and adult GR epidermal KO mice showed only mild skin defects (71). These data indicate that besides being the target of a treatment for psoriasis, keratinocyte-specific loss of GR is involved in the etiopathogenesis of the disease.

In control adult mouse skin, treatment with imiquimod strongly downregulated *Nr3c1* as well as the closely related mineralocorticoid receptor (MR/*Nr3c2*), which also plays anti-inflammatory roles in this tissue and can be activated by GCs (69, 72–74). Accordingly, GR- or MR- epidermal KO adult mice displayed increased susceptibility to imiquimod-induced psoriasis, and the loss of both receptors had significantly higher impact on disease severity (72). In the absence of epidermal GR and/or MR, regulation of downstream targets, like *Gilz*, is affected. In cultured keratinocytes, *Gilz* was induced by GCs in a GR-dependent manner (16, 17), consistent with a GR-ChIP sequencing experiment that identified GR-binding sites downstream of the *Tsc22d3* gene (17). Importantly, full induction of *Gilz* in response to GCs requires the presence of both GR and MR and GC-induced binding of GR to the genomic binding site near *Tsc22d3* was diminished in the absence of MR (17, 73).

**Figure 1:**
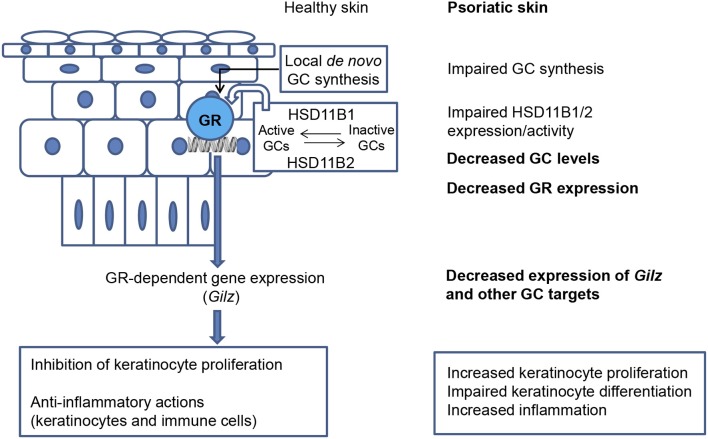
Defective cutaneous GC signaling in psoriasis. Healthy skin is able to synthesize and release GCs *de novo* by a hypothalamic–pituitary–adrenal axis analog. The interconversion between inactive and active GCs by the enzymes 11-beta hydroxysteroid dehydrogenases type 1 and 2 (HSD11B1/HSD11B2) provides another source of corticosteroids. When local steroidogenesis is stimulated, GC-activated GR regulates gene expression, including that of *Gilz*. The actions of GCs in skin limit proliferation and inflammation. In psoriatic skin, *de novo* synthesis of GCs is strongly decreased and the expression/activity of HSD11B1/2 is impaired; decreased GC levels have an overall negative impact on epidermal differentiation. The downregulation of GR and downstream anti-inflammatory mediators in psoriatic lesions likely aggravates disease severity, including increased keratinocyte proliferation, impaired keratinocyte differentiation, and increased inflammation.

In agreement with mouse models, expression of GR, MR, and GILZ (33, 34, 75, 76) was downregulated in human psoriatic lesions (Figure 1). Also, it has been reported that GR nuclear translocation was reduced in psoriatic skin (77, 78). Importantly, *GILZ* expression negatively correlated with levels of pro-inflammatory cytokines IL-17A, IL-23, and IL-22; and STAT3 in psoriatic lesions (33). In mice and humans, the expression of other GC-target genes such as *ZFP36, FKBP51*, and *ZBTB16* was also decreased in psoriasis (34, 72), likely aggravating disease severity. The findings that ZFP36 destabilizes *GILZ* mRNA suggests a mechanism by which GILZ levels are fine-tuned following exposure to GCs or cytokines that regulate these genes (44).

### Defective Cutaneous Glucocorticoid Signaling in Psoriasis

Healthy skin is able to synthesize and release GCs through its own local HPA axis analog (Figure 1); however, the pathological relevance of local GC production had not been addressed until recently (4, 75, 76, 79, 80). In line with the observation that GC-target genes are downregulated in psoriasis, metabolomics and transcriptomic profiling demonstrated that cortisol was amongst the most decreased compounds in psoriatic vs. non-lesional skin (76). It was also shown that *de novo* synthesis of GCs was strongly decreased in psoriatic skin lesions (Figure 1) due to reduced expression of steroidogenic enzymes including steroidogenic acute regulatory protein (StAR), 3β-Hydroxysteroid dehydrogenase (3bHSD1), and the cytochrome P450 proteins CYP11A1, and CYP17 (75). 11-beta hydroxysteroid dehydrogenases type 1 and 2 (HSD11B1/HSD11B2) are responsible for cortisol to cortisone interconversion (81). Their expression ratio and activity is important for modulating epidermal differentiation, and have been reported to be altered in lesional tissue [Figure 1; (75, 76)]. Consistent with this, treatment with TNF-α, IL-17A, and IL-22 cytokines suppressed *HSD11B1* and *HSD11B2* expression in human keratinocytes in a reconstituted skin model (76).

Importantly, psoriatic patients that received topical GCs treatments not only normalized epidermal differentiation and skin inflammation but also restored endogenous GC biosynthesis in this tissue (76). Strikingly, mice exposed to clinically relevant doses of UVB showed induction of the systemic steroidogenic pathway, including GC production, indicating communication between the skin, and central HPA axes (82). This could explain at least partially why UVB therapy is beneficial for psoriatic patients and indicates that systemic and local GC levels are vital for cutaneous homeostasis. Altogether, these findings support that defective GC signaling in the skin (by keratinocytes and likely other cell types) is involved in the etiopathogenesis of psoriasis as it interferes with epidermal differentiation, eliciting sustained inflammatory responses. In this scenario, restoration of normal GC signaling represents one major objective, underscoring the relevance of elucidating the specific role of GILZ in psoriasis.

## GILZ and Mouse Models of Psoriasis

**Figure 2:**
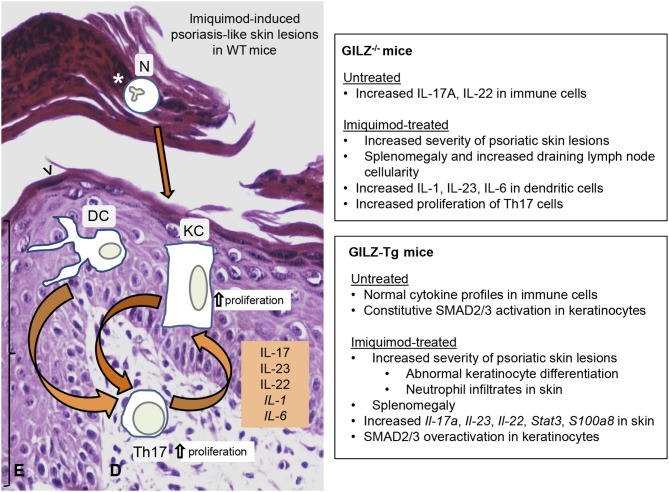
Impact of GILZ in skin psoriatic lesions: Phenotypes of GILZ^−/−^ and GILZ-overexpressing mice. **(Left)** Cell type-specific contributions to cutaneous alterations in psoriatic lesions induced by the imiquimod mouse model in WT mice. Epidermal thickening (bracket), abnormal differentiation of keratinocytes (arrowhead), and intra-epidermal neutrophil infiltrates (asterisk) are indicated. Dysregulation of both immune cells and keratinocytes leads to cytokine overproduction, resulting in immune infiltrates, epidermal hyperproliferation, and abnormal epidermal differentiation. Arrows represent communication between cell types. **(Right)** Summary of phenotypes in GILZ^−/−^ (34) and GILZ-overexpressing (GILZ-Tg; 18) mice. Briefly, while untreated GILZ^−/−^ mice had increased IL-17A and IL-22 in immune cells, both GILZ^−/−^, and GILZ-Tg treated mice showed increased severity of imiquimod-induced psoriatic lesions. GILZ-Tg keratinocytes had constitutively increased phosphorylation of SMAD2/3, which was further increased by imiquimod. E, epidermis; D, dermis; N, neutrophils; DC, dendritic cells; KC, keratinocytes.

The role of GILZ in the imiquimod-model of psoriasis was evaluated using gain- and loss-of-function mouse models (Figure 2). In control mice, besides the cutaneous phenotype, topical imiquimod also induces systemic effects including increased circulating cytokines and splenomegaly (61). While detailed histological evaluation of GILZ^−/−^ skin has not been published, GILZ^−/−^ mice treated with imiquimod showed increased severity in disease parameters, including the macroscopic skin phenotype of scaling and swelling; pro-inflammatory cytokine production; splenomegaly, and draining lymph node cellularity (33). The higher susceptibility to imiquimod-induced inflammation in GILZ^−/−^ mice was explained by the augment of Th17-inducing cytokines by dendritic cells (IL-1, IL-23, and IL-6), and increased proliferation of Th17 cells (33). However, it is important to note that untreated GILZ^−/−^ mice have increases in IL-17A and IL-22 producing lymphocytes and that the contribution of these basal alterations to the disease elicited in the psoriasis model is unclear. Importantly, while addition of IL-6 to Th17-promoting cytokines IL-1β/23 increased T cell proliferation and expression of Th17 genes *in vitro*, exogenous delivery of GILZ restored regulation of Th17 cell proliferation (33). These data confirm that GILZ is key to restrict pathogenic Th17 responses, which may be relevant for psoriasis treatments (23, 33).

Given the role of GILZ in suppressing Th17 responses and its downregulation in psoriatic lesions (33, 34), it was expected that transgenic mice with generalized overexpression of GILZ [GILZ-Tg mice (18)] would be protected from imiquimod-induced inflammation. Surprisingly, these animals showed a dramatic increase relative to controls in many disease parameters, including splenomegaly, and increased number and severity of skin lesions. GILZ-Tg mice showed increased scaling, abnormal keratinocyte differentiation, neutrophil infiltrates, and increased induction of molecules associated with the human disease (*Il-17, Il-22, Il-23, Il-6*, and *Stat*3). However, the systemic response to imiquimod was similar in GILZ-Tg and control mice (as was also the case in the cecal ligation, and puncture sepsis protocol in GILZ-Tg mice; (14), and there were not significant differences in the composition of skin neutrophil or T cell infiltrates of GILZ-Tg vs. controls (18).

Also, the pro-inflammatory actions of GILZ overexpression were specific to skin as neither intestine nor spleen showed increases in Th17-dependent cytokines relative to controls. The deleterious effects of GILZ in the psoriasis model were likely due to its overexpression in epidermis, rather than immune cells, as TGF-β1 signaling via SMAD2/3 was constitutively activated in GILZ-Tg keratinocytes. Moreover, GILZ overexpression in cultured keratinocytes enhanced the induction of the psoriatic marker *S100a*8 in response to IL-17A (18). Similar to human disease, imiquimod-treated control skin showed reduced *Gilz* expression (18). In contrast, as *Gilz* was not downregulated in GILZ-Tg skin, it is feasible that the resolution of inflammation requires reduced levels of GILZ, and that continuous expression, and/or relatively high levels of this GC-target gene can exert pro-inflammatory actions.

## Conclusion

Despite their efficacy, topical administration of GCs to psoriatic patients is accompanied by adverse effects including loss of skin barrier function and increased susceptibility to inflammation and infections. Also, later stages of inflammatory diseases are characterized by a vicious circle of decreased response to GCs, resulting in lower production of anti-inflammatory mediators like GILZ and further loss of control of inflammation. Given these limitations, there is need of improving GC-based therapies for psoriasis and the delivery of GILZ appears as an attractive possibility. There is an inverse correlation between GILZ expression and psoriatic lesions; however, it is unclear whether lower levels aggravate the disease or are part of the pathogenesis. Also, the findings in mice that both gain- and loss of function of GILZ result in higher susceptibility to imiquimod-induced psoriasis raise questions about the therapeutic potential of exogenous GILZ for this skin pathology. Apparent discrepancies may derive from yet uncharacterized cell-type specific functions of GILZ such as recently reported effects on neutrophil and macrophage phagocytosis modulating bactericidal activity. Also, as an exon common to all isoforms of *Tsc22d3* was deleted in GILZ^−/−^ mice, it is plausible that other GILZ isoforms play differential roles. Above threshold effects from overexpression in GILZ-Tg mice may also explain these seemingly controversial results. Until the physiological role of GILZ in all skin compartments is better understood, therapies based on generalized delivery of GILZ seem premature. Based on the relevance of cutaneous GC-signaling, one may speculate on future strategies of local delivery of GILZ specifically to immune cells. It is also feasible that in psoriasis, the ability to produce GILZ in response to GCs could be used to stratify patients into two groups: those who upregulate this GC target would be good candidates for GC therapy and those who do not could be candidates for GILZ delivery to bypass the resistance.

## Author Contributions

LS and PP wrote the manuscript and approved this version for publication.

### Conflict of Interest Statement

The authors declare that the research was conducted in the absence of any commercial or financial relationships that could be construed as a potential conflict of interest.

## Abbreviations

GC: glucocorticoid
GR: glucocorticoid receptor
HPA: hypothalamic–pituitary–adrenal
GILZ: Glucocorticoid-Induced-Leucine-Zipper
MAPK: mitogen-activated protein kinase
TGF-β: transforming growth factor beta
TNF-α: Tumor necrosis factor alpha
IFN: interferon
ROR-γ: retinoic acid-related orphan receptor gamma
ZFP36/TTP: Zinc Finger Protein 36/TrisTetraProlin.
